# Supplementary material for: Content-rich biological network constructed by mining PubMed abstracts
Source: BMC Bioinformatics. 2004 Oct 8;5:147. doi: 10.1186/1471-2105-5-147 (PMC528731; doi:10.1186/1471-2105-5-147)
Supplement: Additional File 5 — The original Chilibot query results of the term "long-term potentiation (LTP)" and 22 other terms, limiting the latest references analyzed to the years 1990, 1995, 2000, and 2004. [file 1471-2105-5-147-S5.bz2 › chilibotAdditionalFile5/ltp2004/html/CREB_ATF.html]

 


 **CREB** and **ATF** 
  
Found 523 abstracts in PubMed,  **30 abstracts were retrieved and analyzed**.  


---

 Search Google  |
 PDF files only 
|  EDU domain only 

---

**Interactive relationship** (e.g. stimulation, inhibition, etc)

- GF 109203X and calphostin C inhibitors of protein kinase C blocked the effect of glucosamine on ANG gene expression and  **CREB**  phosphorylation, but had no impact on p38 MAPK and  **ATF**  2 phosphorylation.  Ref: 12960040 Endocrinology, 2003
- The expression of the proglucagon gene has been demonstrated to be regulated by a cAMP dependent pathway through cAMP response element binding protein  **CREB**  and possibly other transcription factors bound to its cAMP response element CRE activated transcription factor  **ATF**  site.  Ref: 12815047 J Biol Chem, 2003

- :-)

  **Parallel relationship** (e.g. studied together, co-existance, homology, etc.)

  - The ICER proteins contain DNA binding leucine zipper domains that make them endogenous inhibitors of transcription driven by  **CREB**  cAMP responsive element binding protein and its cognates, CREM and  **ATF**  1 activating transcription factor 1.  Ref: 14713288 J Neurochem, 2003
  - Transcription factors, such as the mammalian  **ATF**   **CREB**  family of transcriptional regulators, have diverse functions in controlling cell proliferation and apoptosis.  Ref: 12766482 Apoptosis, 2003
  - SB 203580 a specific p38 MAPK inhibitor attenuated glucosamine action on ANG gene expression as well as p38 MAPK and  **ATF**  2 phosphorylation, but not that of  **CREB** .  Ref: 12960040 Endocrinology, 2003
  - A rhIL 17 dependent transcriptional pulse 1.76 0.11 fold induction was initiated by  **ATF**  2  **CREB**  1 transactivation through the  **ATF**  CRE enhancer site in the proximal promoter.  Ref: 12746433 J Biol Chem, 2003
  - The role of  **ATF**   **CREB**  family members in cell growth, survival and apoptosis.  Ref: 12766482 Apoptosis, 2003
  - We also demonstrate that the  **ATF**   **CREB**  site may cooperatively interact with the NRSF like element in regulating sFrp4 promoter activity.  Ref: 12974383 Biol Chem, 2003
  - In contrast, Tax normally activates the  **CREB**   **ATF**  pathway leading to full stimulation of an HTLV I LTR reporter construct in IKK1 cells.  Ref: 14963024 J Biol ChemJ Biol Chem, 2003
  - This observation was also supported by findings that curcumin did not inhibit UV induced phosphorylation of  **CREB**   **ATF**  1 and  **ATF**  2.  Ref: 14674708 Mol Cell Biochem, 2003
  - Exposure to forskolin, which resulted in  **CREB**  and  **ATF**  1 phosphorylation, caused a reversible decrease in cell volume 14.5 5%.  Ref: 14667824 Exp Eye Res, 2004
  - Tax p40Tax encoded by the env pX gene of human T cell leukemia virus type I HTLV I interacts with cyclic adenosine monophosphate response element binding protein activation transcription factor 1  **CREB**   **ATF**  1 transcription factors of host cells and activates the viral long terminal repeat LTR promoter.  Ref: 12782019 Exp Mol Pathol, 2003
  - Transcriptional regulation of the murine brca2 gene by  **CREB**   **ATF**  transcription factors.  Ref: 14680822 Biochem Biophys Res Commun, 2003
  - This integration appears to require intermediate steps, including expression of pitx genes, and members of the  **ATF**   **CREB**  and Ets transcription factor families.  Ref: 12815727 Bioessays, 2003
  - Glucosamine stimulated ANG and renin mRNA expression and enhanced p38 MAPK,  **ATF**  2, and  **CREB**  phosphorylation in normal glucose 5 mm medium.  Ref: 12960040 Endocrinology, 2003
  - Induction of TGF beta 1 synthesis by 30 mmol D glucose was associated with induction of PKC alpha, phosphorylation of cAMP responsive element binding protein  **CREB**  and activating transcription factor 1  **ATF**  1, and increased fibronectin and type I collagen translation.  Ref: 12846747 Kidney Int, 2003
  - Electrophoretic mobility shift assays demonstrated that NF kappaB binding activity induced by IL 1beta or LPS was also increased in K rasAsp12 transfected cells, along with the binding of  **CREB**  1, CREM 1,  **ATF**  1,  **ATF**  2, and Jun D to a cAMP responsive element CRE like site and the binding of C EBPbeta to a C EBP binding consensus site.  Ref: 14576830 Oncogene, 2003
  - The results of electrophoretic mobility shift assay EMSA showed high levels of NF kappaB,  **CREB**   **ATF**  1, and SRF specific DNA binding activity in all Tax positive cell lines.  Ref: 12858414 J Med Virol, 2003
  - We find that expression of activating transcription factor 3 ATF3, a member of the  **ATF**   **CREB**  subfamily of basic region leucine zipper bZIP proteins, is induced in response to endoplasmic reticulum ER stress or amino acid starvation by a mechanism requiring eIF2 kinases PEK Perk or EIF2AK3 and GCN2 EIF2AK4, respectively.  Ref: 14729979 Mol Cell Biol, 2004
  - this nucleotide sequence contains one putative  **CREB**   **ATF**  consensus site cAMP responsive element CRE.  Ref: 14680822 Biochem Biophys Res Commun, 2003
  - Herein, we show that cadmium induces reactive oxygen species ROS that activate c Jun N terminal kinase JNK and p38 mitogen activated protein kinase MAPK and their substrates, activating transcription factor 2  **ATF**  2, CRE binding protein  **CREB**  and c Jun.  Ref: 14687664 Cell Signal, 2004
  - Using an inducible system Jurkat cell line JPX 9, a dramatic increase in NF kappaB,  **CREB**   **ATF**  1, SRF, and SP1 binding activity, as well as productive HCMV infection, were observed upon Tax expression.  Ref: 12858414 J Med Virol, 2003
  - One particular  **ATF**   **CREB**  family member, ATFx, is an anti apoptotic factor that plays an essential role in cell survival.  Ref: 12766482 Apoptosis, 2003
  - **CREB**   **ATF**  defective Tax mutant M47 activated the p21 promoter significantly less efficiently.  Ref: 14520699 Int J Cancer, 2003
  - The transcription factors CREM,  **CREB** , and  **ATF**  1 constitute a subfamily of beta Zip transcription factors.  Ref: 14654248 Mol Cell Endocrinol, 2003
  - To achieve this goal, real time RT PCR was used to quantify the mRNA level of several different transcription factors YY1, HMGA1, RY 1, P300,  **CREB** , YAP65, HMGN1, HMGB1, NFAR, OCT 4, TEAD2,  **ATF**  1, HMGN2, MSY2 and TBP in germinal vesicle GV and metaphase II MII bovine oocytes and in, 8 cell and blastocyst stage embryos produced in vitro.  Ref: 14960490 Biol ReprodBiol Reprod, 2003
  - Elsewhere we have shown that ATF3, a member of the  **ATF**   **CREB**  subfamily of the basic leucine zipper domain proteins, is expressed predominantly in the alpha cells of the pancreatic islets.  Ref: 12815047 J Biol Chem, 2003
  - It may be speculated that further downstream signals include activation of transcription factors such as nuclear factor kappa B NF kappaB, activating transcription factor 2  **ATF**  2 or cAMP response element binding protein  **CREB** .  Ref: 14587531 Int J Occup Med Environ Health, 2003
  - Cloning and characterization of a novel member of the human  **ATF**   **CREB**  family ATF2 deletion, a potential regulator of the human DNA polymerase beta promoter.  Ref: 12909347 Gene, 2003
  - This chromatin structure alteration required components for the stress activated protein kinase SAPK cascade and both subunits of the M26 binding  **CREB**   **ATF**  type protein Atf1.Pcr1.  Ref: 14762213 Nucleic Acids Res, 2004
  - In addition to sites 1 and 2, putative transcriptional factor binding sites for TFIID, SP1 GC and  **ATF**   **CREB**  exhibited positive, while the site for NRSE exhibited negative regulatory functions, as determined by the alkaline phosphatase activities of the reporter assay.  Ref: 12974383 Biol Chem, 2003
  - Elevation of cAMP in response to forskolin was confirmed by determining the phosphorylation of cAMP response element binding protein and activating transcription factor 1  **CREB** ,  **ATF**  1, which form the downstream targets of protein kinase A.  Ref: 14667824 Exp Eye Res, 2004
  - Taken together, these findings suggest that HTLV 1 Tax activates p21 Waf1 Cip1, a cell growth inhibitor, in a p53 independent mechanism through  **CREB**   **ATF**  related transcription factors, and inhibits cdk2.  Ref: 14520699 Int J Cancer, 2003
  - In the present study, we examined the expression of another member of the  **CREB**   **ATF**  family of transcription factors, ATF2, in the brains of rats chronically treated with two different antidepressants, and in rats 4 months after their exposure to prolonged stress.  Ref: 14647483 NeuropsychopharmacologyNeuropsychopharmacology, 2003
  - It comprises the X1, X2 and Y boxes, bound by RFX, X2 BP  **ATF**   **CREB**  and NFY factors, respectively.  Ref: 14678199 Immunology, 2004
  - ATF2, A Member of the  **CREB**   **ATF**  Family of Transcription Factors, in Chronic Stress and Consequent to Antidepressant Treatment Animal Models and Human Post Mortem Brains.  Ref: 14647483 NeuropsychopharmacologyNeuropsychopharmacology, 2004
  - ATF3 Activating transcription factor 3, a member of the  **CREB**   **ATF**  family, can be induced by stress and growth factors in mammalian cells, and is thought to play an important role in the cardiovascular system.  Ref: 14723708 Genes CellsGenes Cells, 2004
  - Transcription of the fission yeast fbp1 gene and recombination at the meiotic recombination hotspot ade6 M26 M26 are both regulated by cAMP responsive element CRE like sequences and the  **CREB**   **ATF**  type transcription factor Atf1\*Pcr1.  Ref: 14573465 Genetics, 2003
  - **CREB** , CREM, and or  **ATF**  1 functionally bound to and regulated this promoter.  Ref: 14680822 Biochem Biophys Res Commun, 2003
  - Thus,  **ATF**  2 regulates chondrocyte maturation as a direct target of TGF beta signaling while  **CREB**  regulates differentiation by targeting genes independent of the individual signaling effects of TGF beta or BMP 2.  Ref: 12878171 Exp Cell Res, 2003
  - Phosphorylation of p38 MAPK, activating transcription factor 2  **ATF**  2, and cAMP responsive element binding protein  **CREB**  was determined by Western blot analysis.  Ref: 12960040 Endocrinology, 2003
